# Optimizing Neuropsychological Assessments for Cognitive, Behavioral, and Functional Impairment Classification: A Machine Learning Study

**DOI:** 10.1155/2017/1850909

**Published:** 2017-01-31

**Authors:** Petronilla Battista, Christian Salvatore, Isabella Castiglioni

**Affiliations:** Institute of Molecular Bioimaging and Physiology, National Research Council (IBFM-CNR), Segrate, Milano, Italy

## Abstract

Subjects with Alzheimer's disease (AD) show loss of cognitive functions and change in behavioral and functional state affecting the quality of their daily life and that of their families and caregivers. A neuropsychological assessment plays a crucial role in detecting such changes from normal conditions. However, despite the existence of clinical measures that are used to classify and diagnose AD, a large amount of subjectivity continues to exist. Our aim was to assess the potential of machine learning in quantifying this process and optimizing or even reducing the amount of neuropsychological tests used to classify AD patients, also at an early stage of impairment. We investigated the role of twelve state-of-the-art neuropsychological tests in the automatic classification of subjects with none, mild, or severe impairment as measured by the clinical dementia rating (CDR). Data were obtained from the ADNI database. In the groups of measures used as features, we included measures of both cognitive domains and subdomains. Our findings show that some tests are more frequently best predictors for the automatic classification, namely, LM, ADAS-Cog, AVLT, and FAQ, with a major role of the ADAS-Cog measures of delayed and immediate memory and the FAQ measure of financial competency.

## 1. Introduction

Dementia is a clinical syndrome which affected more than 35 million people worldwide in 2010, with new estimates of 48.1 million people for 2020 and numbers expected to almost double every 20 years [1]. Alzheimer's disease (AD) represents the primary cause of neurodegenerative dementia [2].

To date, scientists have concentrated on untangling the complex brain changes involved in the onset and progression of AD. However, this pathology is correlated to cognitive impairment, behavioral disturbance, and functional disabilities, which greatly have an impact on the quality of daily life, and is major problem for families, caregivers, and healthcare institutions. It is thus crucial to detect such changes early and to identify the level and the type of impairment in the patients. This could facilitate the provision of optimal support as soon as possible, in order to maintain their quality of life for as long as possible. In addition, early detection enables the disease to be monitored from its initial stage of disability, possibly administering available treatments when loss of functions is not yet advanced.

Neuropsychological assessment plays a crucial role in detecting loss of cognitive functions and change in behavioral and functional state from normal conditions. Specifically, neuropsychological tests can detect dysfunctions in human “cognitive domains” as a consequence of dysfunctions in different neural networks and subnetworks caused by AD. In 2013, the American Psychiatric Association published the fifth edition of the Diagnostic and Statistical Manual of Mental Disorders (DSM-5) [3]. DSM-5 defined six key domains of cognitive function, namely, complex attention, executive function, learning and memory, language, perceptual-motor function, and social cognition, and each of these has subdomains. Identifying the domains and subdomains affected in a patient helps in establishing the aetiology and severity of the neurocognitive disorder. Neuropsychological tests can measure different cognitive domains (e.g., language, learning, and memory) and subdomains (e.g., long-term memory and recognition memory) [4]. However, despite the promising results from several different tests, identifying the best ones, as well as the best combination of tests to be used to classify and diagnose AD, is still a matter of debate, and a large amount of subjectivity continues to exist in the diagnostic process [5, 6]. In fact, even the DSM-5 does not name any proprietary tests. In addition, whether specific tasks are better for detecting impairment than others is still unclear [7]. A long list of neuropsychological tests is thus still considered appropriate and subjects are addressed to intensive testing. Optimizing or even reducing the amount of neuropsychological tests used to classify AD patients, also at an early stage of impairment, may be possible with no additional performance costs, thus reducing the time intensity and cognitive stress of the assessment.

Machine learning (ML) is an advanced computational technique that can be used for automatic classification of subjects with diagnostic purpose. Specifically, methods based on ML are able to learn the relationship between input and response variables of two given classes of subjects (e.g., normal and pathological subjects) and to use the learned model to predict the response variable of a new (independent) subject. ML was first adopted in medicine because of its ability to deal with large and complex datasets [8].

Sophisticated ML methods have been applied in the field of dementia in order to obtain a high level of accuracy in the automatic classification of subject impairment [9]. However, such methods have been extensively used with neuroimaging studies on dementia (e.g., [10]) and there have been few explicit attempts to use ML to assess cognitive, behavioral, and functional measures.

In 2015, Weakley et al. [11] used ML and a combination of twenty-seven measures from a cohort of 272 subjects including cognitive, behavioral, and functional abilities obtained from different neuropsychological tests (e.g., visual and verbal memory and language category fluency) to automatically classify groups of patients with different clinical dementia ratings (CDR), namely, CDR = 0, CDR = 0.5, and CDR = 1+ (i.e., 1 and 2). The CDR Scale is a five-point semistructured interview between the patient and a reliable informant (e.g., caregivers) designed to stage the severity of dementia considering the state of the subject with respect to memory, orientation, judgment and problem solving, community affairs, home/hobbies, and personal care [12]. Weakley et al. [11] envisaged the potential of ML with respect to traditional statistical approaches in fully automating the diagnostic process by reducing time-consuming and subjective manual analyses and producing reliable information on the relationship between input (measures of cognitive, behavioral, and functional abilities) and response variables (CDR score) without the need of defining assumptions on data [13]. The authors used ML (with respect to manual classification) to explore many measure configurations (i.e., many combinations of measures), which was impossible to analyze manually. The ML classifier selected a maximum of six measures able to predict the CDR score (an accuracy of 98%, 82%, and 94% was obtained when classifying CDR = 1+ versus CDR = 0, CDR = 0.5 versus CDR = 0, and CDR = 1 versus CDR = 0.5, resp.). The results showed that ML was a stable and robust predictive model for a number of approximately 200 participants.

A similar approach was already reported by another study in the literature [14], in which ML was applied to only seven cognitive and behavioral attributes from a database of 765 subjects (together with their educational level and the clinical estimation of the patient insight) to predict CDR scores (0, 0.5, 1, 2, and 3). However, these attributes were selected by the authors and leaded to poor classification accuracy for the mildly demented severity class (59%).

Given the results obtained by the two above-mentioned studies, it would be interesting to consider the use of more subdomains for the automatic classification, since it could lead to an improvement in the predictive model and classification performance.

In this work, our aim was to assess the potential of automatic classification in optimizing or even reducing the amount of neuropsychological measures to predict cognitive, behavioral, and functional impairment of subjects with AD, even at an early stage. We investigated the role of twelve state-of-the-art neuropsychological tests in the automatic classification of subjects with none, mild, or severe impairment as measured by CDR. Data were obtained from the ADNI database (http://adni.loni.usc.edu/). In the groups of measures used as classification features, we included measures of both cognitive domains (language, executive function, memory and learning, and complex attention) and subdomains (perceptual-motor coordination, working memory, and visuoconstructional reasoning). We also included measures of depression and loss of awareness, more specifically, orientation. In order to reduce the dimensionality of the computation, we used the following two approaches: (1) an automated classification following a computational feature reduction method and (2) an automated classification following a feature reduction guided by neuropsychologists.

## 2. Materials and Methods

### 2.1. Participants

Data used in the preparation of this article were obtained from the Alzheimer's Disease Neuroimaging Initiative (ADNI) database (http://adni.loni.usc.edu/). The ADNI was launched in 2003 as a public-private partnership, led by the principal investigator, Michael W. Weiner, MD. The primary goal of ADNI was to test whether serial magnetic resonance imaging (MRI), positron emission tomography (PET), other biological markers, and clinical and neuropsychological assessments can be combined to measure the progression of mild cognitive impairment (MCI) and early Alzheimer's disease (AD); see http://www.adni-info.org/.

In our study, a total of 324 subjects were considered. This sample is the same as in our previous study [10].

All subjects performed a CDR assessment at follow-up (from at least 18 months to 36 months). The interview included both answers directly obtained from each subject and from the interviewer observing the subjects when performing short and easy tasks. The CDR total score came from the subscores of measures on memory, orientation, capacity of judgment/problem solving, social and business activities, capacity to make home-life/intellectual activities/hobbies, and personal care. In our work, the CDR score served as a gold standard to classify each participant into one of the following three categories: absence of impairment with CDR = 0, mild impairment with CDR = 0.5, and severe impairment with CDR = 1. In each category, subjects were matched for age and gender. 126 subjects had CDR = 0 at follow-up, 143 subjects had CDR = 0.5, and 55 subjects had CDR = 1 at follow-up.

### 2.2. Measures Used as Features

All subjects underwent a neuropsychological assessment, starting from the screening visit (visit at time = 0 of the ADNI protocol) up to the baseline (visit at time = 1 month from the screening in the ADNI protocol) and following visits. The ADNI database provided the raw results of this assessment in terms of total and partial test scores.

In our work, we used both total and partial test scores as measures to predict the classification category of subjects.

In order to ensure the independence among features and the gold standard (CDR), we excluded the CDR test from the neuropsychological measures used as features. This avoided circularity in the classification process, thus reducing overfitting.

In the next sections, we present a brief description of the neuropsychological tests and relative measures used as features.

#### 2.2.1. Mini Mental State Examination (MMSE)

MMSE is a brief questionnaire developed by Folstein et al. [15] which measures the global cognitive impairment and takes around 15 minutes to complete. It consists of 30 items divided into 6 areas: orientation in time and space; memory (repetition of three words), attention and calculation (serial subtraction or forward/backward spelling, recall of words previously memorized); language (recognition of two objects, repetition of a short sentence; sentence comprehension; sentence writing), and constructional praxis (design copy). In our study we used all of the 32 measures reported in the ADNI database: MMDATE, MMYEAR, MMMONTH, MMDAY, MMSEASON, MMHOSPIT, MMFLOOR, MMCITY, MMAREA, MMSTATE, MMBALL, MMFLAG, MMTREE, MMTRIALS, MMD, MML, MMR, MMO, MMW, MMBALLDL, MMFLAGDL, MMTREEDL, MMWATCH, MMPENCIL, MMREPEAT, MMHAND, MMFOLD, MMONFLR, MMREAD, MMWRITE, MMDRAW, and MMSCORE.

#### 2.2.2. Clock Test

Participants are asked to draw a clock and to set the hands to ten after eleven. Scores are assigned if the symmetry of number placement, correctness of numbers, and hand placement are correct. In our study, we used all of the 12 measures reported in the ADNI database: CLOCKCIRC, CLOCKSYM, CLOCKNUM, CLOCKHAND, CLOCKTIME, CLOCKSCOR, COPYCIRC, COPYSYM, COPYNUM, COPYHAND, COPYTIME, and COPYSCOR.

#### 2.2.3. Logical Memory (LM)

This measures declarative/episodic memory by means of a brief story read to the participant who is asked to retell it from memory immediately. The primary measure of performance is the number of story units recalled. The LM is a subtest of the Wechsler Memory Scale-Revised [16] and one of the most widely used clinical measures of memory. In our study, we used all three measures reported in the ADNI database: LIMMTOTAL, LDELTOTAL, and LDELCUE.

#### 2.2.4. Rey Auditory Verbal Learning Test (AVLT)

AVLT is a widely used test of anterograde verbal episodic memory. A list of 15 unrelated words is presented orally to the subject [17]. The test consists of 5 consecutive repetitions in order to learn the unstructured verbal material and then a long delay free recall 30 minutes later to verify if subject acquired the words over the course of the 5 trials. Finally, a yes/no recognition trial follows the delayed recall trial. It is possible to obtain a learning score from AVLT using the difference between the last and the first immediate recall trials. The measures that are usually calculated from the AVLT are learning scores (trial 5 minus trial 1), short and long delay recall, and recognition. In our study, we used all of the 18 measures reported in the ADNI database: AVTOT1, AVERR1, AVTOT2, AVERR2, AVTOT3, AVERR3, AVTOT4, AVERR4, AVTOT5, AVERR5, AVTOT6, AVERR6, AVTOTB, AVERRB, AVDEL30MIN, AVDELERR1, AVDELTOT, and AVDELERR2.

#### 2.2.5. Digit Span (DS)

DS is a test of working memory with two subscales: forward and backward. In the DS forward, the examiner reads a number sequences of increasing length and asks the participant to repeat them. The total score is the number of sequences correctly repeated. In DS backward, the examiner reads a number sequence of increasing length and then asks the participant to repeat each sequence backward. The primary measure of performance is the number of digit sequences correctly reversed. These two tests are included in the Wechsler Memory Scale-Revised [16]. In our study, we used all five measures reported in the ADNI database: DSPANFOR, DSPANFLTH, DSPANBAC, DSPANBLTH, and DIGITSCOR.

#### 2.2.6. Category Fluency Tests (Animals and Vegetables)

This is a widely used measure of the ability to spontaneously generate a set of semantically related words in one minute. The participant is asked to name different examples of a given category and the score is the number of unique examples named. In our study, we used all six measures reported in the ADNI database: CATANIMSC, CATANPERS, CATANINTR, CATVEGESC, CATVGPERS, and CATVGINTR.

#### 2.2.7. Trail Making Test A-B (TMT A-B)

TMT A-B encompasses two trials, A and B [18]. The first part A is a test of psychomotor processing speed and visual scanning. An array of numbers on a page is presented to the subjects and they are instructed to draw lines connecting the numbers in sequential order within the time allowed. The second part B provides cognitive flexibility measures: psychomotor processing speed, visual scanning, and attentional set shifting. An array of numbers and letters is presented to the subjects and they are asked to draw connecting lines while alternating between numbers and letters in sequential order. An additional commonly used measure is the time to completion from parts A and part B minus part A. In our study, we used all six measures reported in the ADNI database: TRAASCOR, TRAAERRCOM, TRAAERROM, TRABSCOR, TRABERRCOM, and TRABERROM.

#### 2.2.8. Boston Naming Test (BNT)

BNT assesses naming ability using 30 items [19]. Participants are asked to name a series of visual stimuli (object images) with different frequencies (ranging from high to low). If subjects are not able to come up with the correct answer, they are provided with a cue. A phonemic cue is provided when the participant can recognize the purpose of the object but cannot retrieve the correct name. In our study, we used all six measures reported in the ADNI database: BNTSPONT, BNTSTIM, BNTCSTIM, BNTPHON, BNTCPHON, and BNTTOTAL.

#### 2.2.9. American National Adult Reading Test (ANART)

ANART is a widely accepted test to estimate premorbid verbal levels of intelligence in dementing individuals. It consists of 50 orthographically irregular English words. Participants are instructed to pronounce each word aloud, beginning at the top of the list and continuing through to the end. In our study, we used the measure ANARTERR reported within the ADNI database.

#### 2.2.10. Alzheimer's Disease Assessment Scale-Cognitive Behavior (ADAS-Cog)

ADAS-Cog is composed of two parts, the noncognitive subscale and the cognitive subscale, and provides a measure index of global cognition. The tests take around 30–40 minutes to administer. Twelve tests are used to evaluate short-term memory (evocation of words; word recognition; learning the instructions of a test); spatial-temporal orientation; language skills (verbal skills, difficulty in naming spontaneous speech, comprehension of spoken language, naming objects and fingers, and execution of commands); praxis; attention and concentration. The rating of the majority of cognitive tests is assigned on the basis of the performance of the patient in each single test, while, in some cases, it is based on clinical estimates carried out by the examiner in the course of conversation and other sessions. The ADAS-Cog scores range from 0, which is equivalent to the absence of problems, to a maximum of 70, which indicates a serious deficit in all tests. For our study, we used all 15 measures reported in the ADNI database: Q1, Q2, Q3, Q4, Q5, Q6, Q7, Q8, Q9, Q10, Q11, Q12, Q14, TOTAL11, and TOTALMOD.

#### 2.2.11. Geriatric Depression Scale (GDS)

GDS is a 30-item self-report assessment used to identify mood changes (i.e., depression) in elderly patients [20]. The examinee has to provide yes/no answers to each item of the GDS. For our study, we used all 16 measures reported in the ADNI database: GDSATIS, GDDROP, GDEMPTY, GDBORED, GDSPIRIT, GDAFRAID, GDHAPPY, GDHELP, GDHOME, GDMEMORY, GDALIVE, GDWORTH, GDENERGY, GDHOPE, GDBETTER, and GDTOTAL.

#### 2.2.12. Functional Assessment Questionnaire (FAQ)

FAQ is as a self-administered functional assessment which provides information on the patient's physical, psychological, social, and role functions. It can be used useful to monitor the patient over time with 0 score corresponding to no impairment and 30 to severely impaired. In our study, we considered all 11 measures reported in the ADNI database: FAQFINAN, FAQFORM, FAQSHOP, FAQGAME, FAQBEVG, FAQMEAL, FAQEVENT, FAQTV, FAQREM, FAQTRAVL, and FAQ total.

Finally, in our work, a total of 131 measures were used.  Table 1 shows the entire list of these measures, a short description of what they represent together with the reference tests.

### 2.3. Feature Normalization

Raw scores and subscores were first normalized as *z*-scores, using the following formula:

(1) $$\begin{array}{c} z\text{-}score=\frac{\left(score-m\right)}{s}, \end{array}$$

where score represents the raw score or subscore of a given test and *m* and *s* represent the mean and standard deviation, respectively, of the raw score of the subjects.

### 2.4. Feature Reduction

Feature reduction was applied in order to reduce the number of features to be classified without losing relevant information, which resulted in an improvement in computational performance. Two different approaches were implemented: (a) a computational approach, based on the mathematical discriminatory power of features among classes, and (b) an approach based on our basic understanding of the redundancy of features. More specifically, in this last approach, our cognitive understanding of the disease guided the model.

*(a) Computation-Based Feature Reduction*. The class discriminatory power of *z*-scored features was estimated in terms of Fisher's Discriminant Ratio (FDR) as follows:

(2) $$\begin{array}{c} FDR=\frac{{\left(\mu_{1}-\mu_{2}\right)}^{2}}{\sigma_{1}^{2}+\sigma_{2}^{2}}, \end{array}$$

where *μ*_*i*_ and *σ*_*i*_^2^ are the mean and the variance of *i*th class, respectively. 

*(b) Feature Reduction Guided by the Neuropsychologists*. Two experienced neuropsychologists were asked to reduce the number of features on the basis of three primary considerations: (1) redundancy (if the same or similar measures are derived from two or more cognitive tests included in the ADNI database: for example, item Q7 of ADAS overlaps with the MMSE items assessing spatiotemporal disorientation); (2) overlap with CDR (if the same or similar measures are present in the CDR interview which is used as a gold standard for the classification: this could produce bias in the classification performance); (3) poor relevance to AD (based on scientific literature).

The features included in our classification fell within the following domains: global cognitive status, orientation, language, executive functioning, memory, praxis, attention, working memory, visuospatial/constructional ability, functional abilities, and depression, as shown in Table 2.

### 2.5. The Machine Learning Classifier

In order to automatically classify subjects into different groups through the considered neuropsychological measures, we used an ML classifier based on methods previously published by our group [21].

#### 2.5.1. The Classification Algorithm

The classification algorithm is based on Support Vector Machines (SVMs) [22], which generate a predictive model to perform binary group separation. The predictive model is designed as a hyperplane computed using a training set of data as input to SVM. This set consists of (1) a vector of samples belonging to two different classes and (2) the corresponding vector of labels, which identifies the class of each sample. During this training phase, SVM computes a hyperplane to separate the samples belonging to the two training classes. This hyperplane can then be used as a predictive model to classify a new sample into one or the other of the two classes. The predicted class *y* for sample *x* can be computed using the following formula:

(3) $$\begin{array}{c} y\left(\;x\;\right)=\overset{N}{\underset{n=1}{\sum }}w_{n}\cdot t_{n}\cdot k\left(\;x,x_{n}\;\right)+b, \end{array}$$

where *N* is the number of samples included in the training set; *w*_*n*_ is a weight assigned by SVM to each sample *n* in the training set during the training phase; *t*_*n*_ is the label of the sample *n* of the training set; *k*(*x*, *x*_*n*_) is a kernel function; and *b* is a threshold parameter.

We used the Matlab platform to implement the SVM classifier. Our code also included algorithms from the biolearning toolbox of Matlab. Classification was performed using both linear and nonlinear kernels for performance comparison, the latter including a quadratic kernel, a Gaussian Radial Basis Function (RBF) kernel with default sigma = 1, and a Multilayer Perceptron kernel with default scale $\left[\begin{array}{cc} 1 & -1 \end{array}\right]$. For each subject, the CDR score was used as a label for all classifiers.

#### 2.5.2. Cut-Off on Features

*(a) Computation-Based Features*. *Z*-scored features were sorted in descending order according to their FDR. The 5% features with highest FDR were retained for classification.

*(b) Features Chosen by the Neuropsychologists*. Each of the features chosen by the neuropsychologists was used as input into the classifier, thus obtaining individual feature classification accuracy. Features were sorted in descending order according to their classification accuracy. The top 10 features with the highest accuracy were retained for classification.

#### 2.5.3. Optimization of Features and Evaluation of Performance

An optimization of features was performed in order to find the combination of scores and subscores that return the best performance for the classification of the different groups of subjects.

For all kernels of the classifier, we performed a nested 10-fold Cross Validation (nested CV), which consists in (1) splitting the original dataset into 10 subsets of (possibly) equal size; (2) using 10-1 subsets to perform an inner training and validation loop for the optimization of the features; (3) using the 10th held-out subset to perform an outer test loop for the evaluation of the optimized features. In order to test all subsets, this procedure is then repeated 10 times.

Specifically, for each of the 10 rounds of the nested CV, all possible combinations of the reduced features (scores and subscores) were tested, for the two approaches described in Section 2.4. For each of the 10 rounds of the outer loop, accuracy, sensitivity, and specificity were calculated, and results were averaged across all rounds.

In order to avoid problems arising from the use of class-imbalanced datasets, which could lead to the classifier being trained* more* on one class than the other (in a binary-classification framework), the number of subjects in the two classes was kept balanced in both the training and validation sets.

We also evaluated the classification performance using two specific metrics for imbalanced-domain problems, namely, the GM of the true rates and the Dominance [23, 24]. These were computed as follows:

(4) GM=TPTP+FN·TNFP+TN,Dominance=TPTP+FN−TNFP+TN,

where TP (TN) is the number of true positives (negatives) and FP (FN) is the number of false positives (negatives).

The whole nested CV process was repeated for 100 iterations in order to reduce statistical variability of results. In fact, training and validation may depend on a particular random choice for the pair of training and validation sets, which could lead to a wrong classification performance estimate. The use of an iterative procedure helps to prevent this, because classification performances (as well as score frequencies) are averaged across 100 iterations.

A classification was performed for the following three comparisons: (1) CDR = 0.5 versus CDR = 0, (2) CDR = 1 versus CDR = 0.5, and (3) CDR = 1 versus CDR = 0.5.

#### 2.5.4. Features as Best Predictors

The optimal combination of features was chosen as the one with the maximum accuracy of classification in the inner validation loop. Hence, we obtained an optimal combination of features for each round of 10 and for each iteration of 100, thus 1000 optimal combinations of features.

In order to determine which features were the most important for the classification, we computed the frequency of each feature in all optimal combinations. The features were sorted in descending order according to their frequency. The top 10 features with the highest frequency were shown as the best predictors.

## 3. Results

### 3.1. Feature Reduction

**Figure 1:**
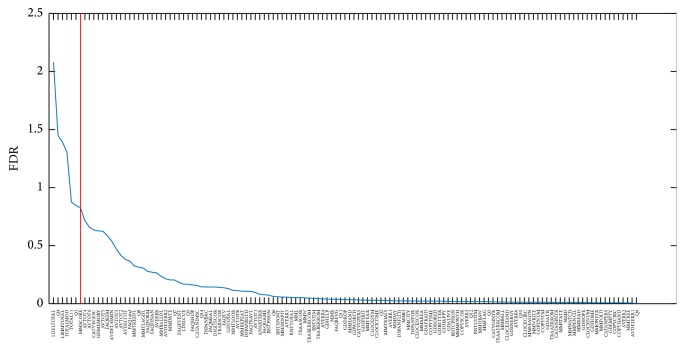
Representative example for one round and one iteration (CDR = 1 versus CDR = 0, round #1, iteration #1) of features ordered according to their FDR.

*(a) Computation-Based Features*.  Figure 1 shows, as representative example for one round and one iteration (CDR = 1 versus CDR = 0, round #1, iteration #1), features ordered according to their FDR. The cut-off is shown reducing the number of features to the 5% with highest FDR, thus reducing the number from 131 to 7 features. Similar figures and results have been obtained for all the other rounds and iterations.

*(b) Features Chosen by the Neuropsychologists*. Table 2 shows the full list of features available from the ADNI database and those chosen by the neuropsychologists (in bold). Features are grouped into cognitive domains. The neuropsychologists reduced the number of features from 131 to 32. The reasons for exclusion are reported.

**Figure 2:**
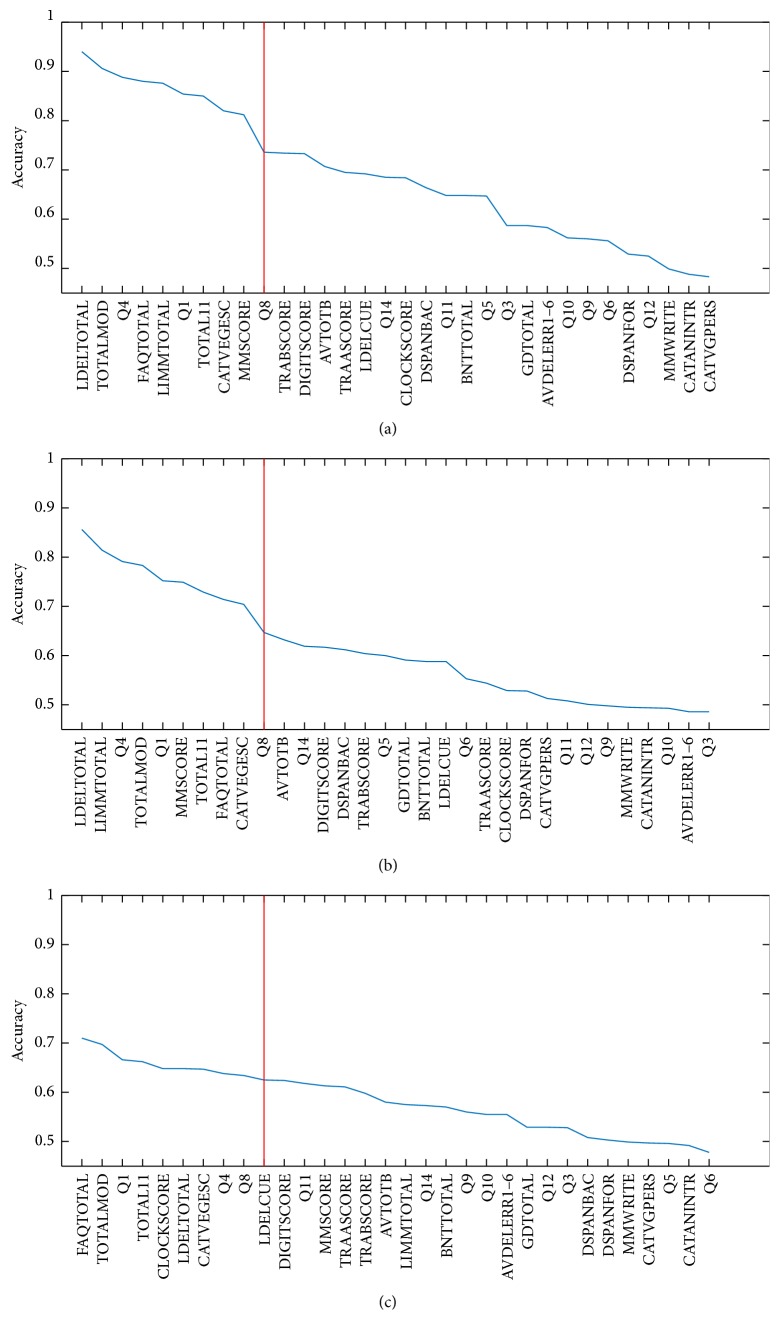
Features ordered according to their individual classification accuracy for CDR = 1 versus CDR = 0 (a), CDR = 0.5 versus CDR = 0 (b), and CDR = 1 versus CDR = 0.5 (c), using the feature reduction guided by the neuropsychologists. Results were obtained as average across all rounds (10) and iterations (100) but for each feature independently. Features are ranked in descending significance with respect to accuracy.

Figure 2 shows the features ordered according to their individual classification accuracy for CDR = 1 versus CDR = 0 (a), CDR = 0.5 versus CDR = 0 (b), and CDR = 1 versus CDR = 0.5 (c). Accuracy was computed as an average over 10 rounds and 100 iterations. The cut-off is shown reducing the number of features to the top 10 with highest classification accuracy, thus reducing the number from 32 to 10 features.

### 3.2. Optimization of Features and Evaluation of Performance

*(a) Computation-Based Features*. The classification performance averaged over all 10 rounds and 100 iterations is shown in Table 3 for CDR = 1 versus CDR = 0, Table 4 for CDR = 0.5 versus CDR = 0, and Table 5 for CDR = 1 versus CDR = 0.5. Accuracy, sensitivity, specificity, Geometric Mean, and Dominance are reported. Each table shows the classification results for linear, quadratic, Gaussian RBF, and Multilayer Perceptron kernels.

Table 6 shows the classification performance in the inner and outer loops of the nested CV for each of the 10 rounds individually. Results (in terms of accuracy of classification) were averaged over all 100 iterations and are shown for CDR = 1 versus CDR = 0, CDR = 0.5 versus CDR = 0, and CDR = 1 versus CDR = 0.5.

As expected, the best classification performance (accuracy, sensitivity, and specificity > 0.89 for the linear kernel) was obtained when discriminating subjects with moderate problems from normal subjects. However, a good performance (accuracy, sensitivity, and specificity > 0.85 for the linear kernel) was also obtained when discriminating subjects with mild impairment from normal subjects. This result is very important for patients, their families, and caregivers, since it suggests that the detection of changes may already be effective at an early stage of impairment. Thus, optimal support may be established and monitored when the cognitive abilities and independence of the subject have not already been compromised.

The most difficult discrimination was between subjects with mild and severe impairments (accuracy, sensitivity, and specificity ranging from 59% up to 67% for the linear kernel). This, however, has a minor impact on patients, their families, and caregivers, since, if early detection of behavioral changes is effective (see our consideration above), the subject may already be managed and monitored as an early dementia patient, and appropriate assistance and any necessary treatment should have already been started.

The classification results using nonlinear kernels (i.e., quadratic, Gaussian RBF, and Multilayer Perceptron kernels) are similar with those obtained using a linear kernel.

*(b) Features Chosen by Neuropsychologists*.  Table 7 shows the classification performance in terms of accuracy, sensitivity, specificity, Geometric Mean, and Dominance averaged across all 10 rounds and all 100 iterations. Results are reported for CDR = 1 versus CDR = 0, CDR = 0.5 versus CDR = 0, and CDR = 1 versus CDR = 0.5.

In Table 8, the classification performance in the inner and outer loops of the nested CV for each of the 10 rounds individually is shown. Accuracy of classification was obtained as average over all 100 iterations. The performance for CDR = 1 versus CDR = 0, CDR = 0.5 versus CDR = 0, and CDR = 1 versus CDR = 0.5 is reported.

As expected, also in this case, the best classification performance (accuracy, sensitivity, and specificity > 0.95) was obtained when discriminating subjects with moderate problems from normal subjects. However, a good performance (accuracy, sensitivity, and specificity > 0.84) was also obtained when discriminating subjects with mild impairment from normal subjects. The most difficult discrimination was distinguishing subjects with mild from moderate impairment (accuracy, sensitivity, and specificity ranging from 67% up to 70%).

**Figure 3:**
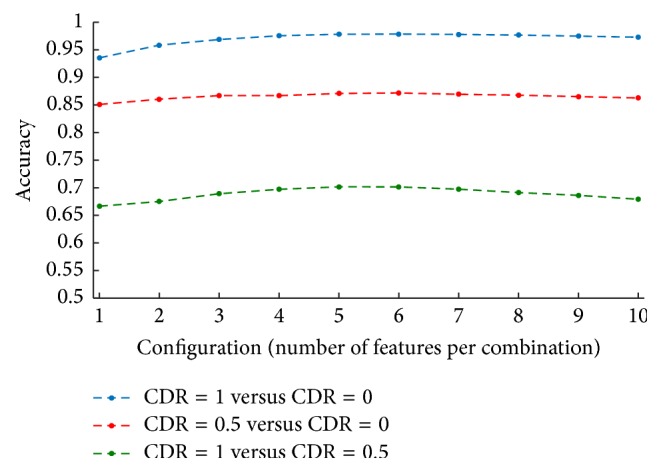
Highest accuracy of classification as a function of the configuration (combination of input features) in the inner loop of the nested CV. Performances are reported for CDR = 1 versus CDR = 0 (blue), CDR = 0.5 versus CDR = 0 (red), and CDR = 1 versus CDR = 0.5 (green), using the features chosen by the neuropsychologists. Results were obtained as average across all rounds (10) and iterations (100).

The trend of the highest accuracy of classification as a function of the configuration (number of features per combination) is shown in Figure 3. The highest accuracy in the inner loop of the nested CV is shown for CDR = 1 versus CDR = 0 (blue), CDR = 0.5 versus CDR = 0 (red), and CDR = 1 versus CDR = 0.5 (green). Results were obtained using the features chosen by the neuropsychologists and averaged across all 10 rounds and all 100 iterations.

As it can be seen, the performance of the model slightly improves when more features are used as input into the classifier, until reaching a plateau at about 5 features. Although the performance of classification is different for each of the three comparisons (CDR = 1 versus CDR = 0, CDR = 0.5 versus CDR = 0, and CDR = 1 versus CDR = 0.5), the trend as a function of the number of features considered is similar.

### 3.3. Features More Frequently Found as Best Predictors

*(a) Computation-Based Features*.  Table 9 shows the top 10 features most frequently found as best predictors across all 10 rounds and all 100 iterations (i.e., most frequently found in the combinations of features with the best classification accuracy) using the FDR feature reduction. Features are ranked by frequency in a descending order. 

*(b) Features Chosen by the Neuropsychologists*.  Table 10 shows the top 10 features most frequently found as best predictors among all 10 rounds and all 100 iterations (i.e., most frequently found in the combinations of features with the best classification accuracy) using the features chosen by the neuropsychologists. Features are ranked by frequency in a descending order.

The two approaches considered for the reduction of features achieved similar results.

In CDR = 1 versus CDR = 0, both approaches found the following features among best predictors: LDELTOTAL, TOTALMOD, LIMMTOTAL, FAQTOTAL, Q4, Q1, and TOTAL11. This is not unexpected, since previous studies have demonstrated that long-term memory reflects the early pathological involvement of the mediotemporal lobe in the course of early AD [25], while functional abilities can occur as a result of cognitive impairment [26].

AVTOT5, AVTOT4, and AVDEL30MIN were found among best predictors when using the FDR-based feature reduction. However, these measures were excluded by the neuropsychologists because of their overlapping with Q1 (Word Recall Task, ADAS) and Q4 (Delayed Word Recall, ADAS) of ADAS.

Q8 and MMSCORE were found among best predictors when using the feature reduction guided by the neuropsychologists. However, these measures were not among the 5% with the highest FDR and thus was excluded in the computation-based classification. MMSCORE has already been found to discriminate early AD from CN with good accuracy (about 70%) [27], while Q8 has been listed among the useful memory and learning tests for AD detection.

In CDR = 0.5 versus CDR = 0, both approaches found the following features as best predictors: LDELTOTAL, Q4, LIMMTOTAL, TOTALMOD, MMSCORE, CATVEGESC, and TOTAL11. Many studies on MCI patients have found an impairment in long-term memory function [28, 29]. The measures that best predict the conversion to AD range from immediate and delayed words recall [30] to verbal fluency. The number of different vegetable names produced is a fluency task that provides measures of language and executive processes, including self-initiated activity, categorization, and mental flexibility that are often involved in MCI patients (e.g., [31, 32]). GDHOPE, MMD, and AVTOT4 were found among the best predictors when using the computation-based feature reduction, but not when using the feature reduction guided by the neuropsychologists, because they were excluded from the list of input features by these neuropsychologists. These measures were excluded because AVTOT4 overlaps with Q1 (Word Recall Task, ADAS), while the other two measures were considered by the neuropsychologists not highly significant in the MCI literature and were not used to further our understanding of cognitive impairment. FAQTOTAL, Q8, and Q1 were found among the best predictors when the feature reduction was based on neuropsychological expertise but not when using the computation-feature reduction, because they were not among the 5% of features with the highest FDR retained for classification. This result is in line with the current literature, since Q1 and Q8 are both measures of long-term memory, and FAQ total has been recently reported as an important feature of MCI [33].

In CDR = 1 versus CDR = 0.5, both approaches found the following features among the best predictors: FAQTOTAL, TOTALMOD, Q1, TOTAL11, CLOCKSCOR, CATVEGESC, and Q8. Most of these features provide information on episodic memory (Q1 and Q8), global cognitive status (the global scores of ADAS and CLOCKSCOR), and language/executive functions (CATVEGESC). Given the primacy of memory in age-related cognitive impairment [34], ML algorithms highlighted measures of episodic memory (recalling information learned previously) as well as recognition memory (a nonverbal task that requires the recognition of the words learned in a longer list of words presented with distracter words). AVTOT5, FAQFORM, and FAQREM were found among best predictors when using the computation-based feature reduction, but they were excluded from the list of input features by the two neuropsychologists. The reason for the exclusion was related to the overlap of AVTOT5 with Q1 (Word Recall Task, ADAS), while the other two measures were considered by the neuropsychologists not highly significant in the MCI/AD literature. LDELCUE, Q4, and LDELTOTAL were found among best predictors when the neuropsychologists guided the feature reduction but not when using the automatic feature reduction because not among the 5% features with the highest FDR retained for the classification. Consistently with the literature, many studies [34–36] have shown anterograde episodic memory as the best marker to predict the conversion to AD in subjects with MCI.

## 4. Discussion 

The ability of neuropsychological measures to help in discriminating between different degrees of cognitive impairment has been widely recognized (e.g., [37, 38]). Despite the existence of clinical measures that are used to classify patients and diagnose clinical disorders showing cognitive impairment, as AD, a large amount of subjectivity affects the diagnostic process. Machine learning is emerging in clinical neuropsychology and neurology as a credible technique to support this process in a quantitative way.

Our work explored the contribution of cognitive, behavioral, and functional measures in the automatic classification of different stages of cognitive impairment using ML. Both total and partial scores as measures of cognitive domains and subdomains from a group of different neuropsychological tests available from the ADNI database were considered as potential predictors of cognitive impairment, even at an early stage.

Our results showed that, among 131 measures considered, it is possible to use a subset of measures in which the classification accuracy is higher than 90% for severe (CDR = 1) versus no impairment (CDR = 0) and higher than 85% for mild (CDR = 0.5) versus no impairment (CDR = 0). Our performances for these two comparisons are similar to those obtained by Logie et al. [6]. These findings could have an impact on the clinical process to perform early diagnosis, with different benefits for patients. However, although we did not include CDR = 2 in the group of patients with severe impairment as Weakley et al. did, accuracy of our classification for patients with CDR = 0.5 versus CDR = 1 was found limited (65–69%) suggesting that our work is in progress and needs improvements. The automatic diagnosis of AD is not challenging and we would expect that ML, by using many features, would be able to predict mild impairment better than subjective techniques. Our model should be modified for this specific comparison in order to achieve best accuracy.

From a methodological point of view, we used a lower number of features than the smallest number of subjects in each group (55 subjects with CDR = 1). This warrants that no curse-of-dimensionality problems occurred. In addition, the feature reduction strategies adopted in our study reduced the number of features used as inputs for the classifier to (at most) 10 (using the feature reduction guided by the neuropsychologists). This number is much lower than the number of subjects in the smallest group, thus avoiding overfitting problems, especially considering the use of SVMs, which are* designed* to handle high-dimensional data.

Concerning the final cognitive profile, when comparing the fully automated classification to the classification guided by the neuropsychologists, a good overlap of results between the two classifications was found. Some tests were found more frequently among best predictors for the automatic classification, namely, LM, ADAS-Cog, AVLT, and FAQ, with a major role of ADAS-Cog measures of delayed and immediate memory and the FAQ measure of financial competency.

There are some measures known to be highly implicated in AD that this model fails to identify. There are some tests that are specifically recommended, that is, the Free and Cued Selective Reminding Test (FCSRT), which is considered as a valid clinical marker for AD [39]. This test was not included in our model since it was not available in the ADNI dataset. These tests provide specific episodic memory measures that correlate with the hippocampal dysfunction, suggesting that a poor performance is typically registered in AD patients [40].

In the field of neurodegenerative diseases, several other studies have applied ML for the identification of subsets of “optimal” classification predictors (e.g., [41, 42]), resulting in a subset of optimal measures, in particular measures of decline in episodic memory. A similar approach has been used for the evaluation of linguistic features within a language test for patients with AD [43–46]. They show the potential of ML in determining the best predictors for language impairment in these patients. Some other studies have investigated this contribution in the classification of AD.

Promising results on similar approaches have been also published in the field of psychiatric disorders. An interesting study by Costafreda et al. [47] showed that pattern recognition algorithms coupled with verbal fluency have a reliable diagnostic power in differentiating schizophrenic patients from healthy controls and patients affected by bipolar disorders. Pina-Camacho et al. [48] used SVM to study predictors of schizophrenia in early-onset first episodes of psychosis. They found that, among the variables included, neuropsychological measures (impaired attention, motor coordination, and global cognition) showed the highest predictive value for a diagnostic outcome of the disease. A study on autism spectrum disorder [49] showed that cognitive measures could be a useful aid to the diagnostic process when assessed by an SVM classifier. However, limitations of these studies were related to the use of cognitive, behavioral, and functional measures during the diagnostic classification of subjects used as a gold standard of analysis, thus inducing overfitting (e.g., [50–52]). In our study, gold standard measures (CDR in our case) were independent from measures used as features. This excluded any potential bias due to circularity in the classification process.

Overall, the results of our work show that ML provides an effective technique to quantify the process to classify patients and to diagnose clinical disorders by using neuropsychological measures. Combined with multicenter databases that collect information from a substantial number of patients (as the ADNI database used in our work), ML was proven to be suitable to conduct statistically meaningful machine learning calculations and to assist clinicians in the optimization of the best measures (and submeasures) to be used, reducing at the same time the subjectivity of the process. For example, we have shown how ML can support the selection of important subscores for the classification of subjects. This could be useful in the optimization of current neuropsychological tests or in the design of new tests for next-generation cognitive assessment.

Notably, cognitive measures found in our works cannot be considered sufficient to perform a neuropsychological classification of patients and we cannot conclude that they are the best predictors from a cognitive perspective, for many reasons. For computation reasons, we have adopted two different strategies to reduce the number of features as input to classification; both strategies have limited the measures used by the machine learning to select the optimal predictors. Furthermore, other measures not included in the considered studies could be behavioral data providing a thorough description of the disease (e.g., loss of empathy, disinhibition, and apathy). These measures could improve the model. In addition, our findings need to be verified in other independent databases and by using other machine learning algorithms, thus proving the generality of our results.

In conclusion, ML approaches can be a useful tool for clinical neuropsychologists especially when they need to deal with a huge amount of data and the level of subjectivity of the process must be reduced. Given that a higher level of accuracy in classification still needs to be achieved and that some questions remain unanswered, further validations and verifications are needed in future research. However, we believe that this study represents a step towards achieving the goal of automatic classification of AD patients by means of clinical neuropsychological assessment.

## Figures and Tables

**Table 1:** List of neuropsychological measures used as features.

Measure	Description
1. MMDATE	What is today's date?, MMSE
2. MMYEAR	What year is it?, MMSE
3. MMMONTH	What month is it?, MMSE
4. MMDAY	What day of the week is it today?, MMSE
5. MMSEASON	What season are we in?, MMSE
6. MMHOSPIT	What is the name of this hospital (clinic, place)?, MMSE
7. MMFLOOR	What floor are we on?, MMSE
8. MMCITY	What town or city are we in?, MMSE
9. MMAREA	What county (district, borough, area) are we in?, MMSE
10. MMSTATE	What state are we in?, MMSE
11. MMBALL	Ball, MMSE
12. MMFLAG	Flag, MMSE
13. MMTREE	Tree, MMSE
14. MMTRIALS	Enter number of trials, MMSE
15. MMD	D, MMSE
16. MML	L, MMSE
17. MMR	R, MMSE
18. MMO	O, MMSE
19. MMW	W, MMSE
20. MMBALLDL	Ball, MMSE
21. MMFLAGDL	Flag, MMSE
22. MMTREEDL	Tree, MMSE
23. MMWATCH	Show the participant a wrist watch and ask “what is this?”, MMSE
24. MMPENCIL	Repeat for pencil, MMSE
25. MMREPEAT	Say “repeat after me: no ifs, ands, or buts.”, MMSE
26. MMHAND	Takes paper in right hand, MMSE
27. MMFOLD	Folds paper in half, MMSE
28. MMONFLR	Puts paper on floor, MMSE
29. MMREAD	Present the piece of paper which reads “CLOSE YOUR EYES,” and say “read this and do what it says”, MMSE
30. MMWRITE	Give the participant a blank piece of paper and say “write a sentence.”, MMSE
31. MMDRAW	Present the participant with the Construction Stimulus page. Say “copy this design.”, MMSE
32. MMSCORE	MMSE total score, MMSE
33. CLOCKCIRC	Approximately circular face, CLOCK
34. CLOCKSYM	Symmetry of number placement, CLOCK
35. CLOCKNUM	Correctness of numbers, CLOCK
36. CLOCKHAND	Presence of the two hands, CLOCK
37. CLOCKTIME	Presence of the two hands, set to ten after eleven, CLOCK
38. CLOCKSCOR	Total score, CLOCK
39. COPYCIRC	Approximately circular face, CLOCK
40. COPYSYM	Symmetry of number placement, CLOCK
41. COPYNUM	Correctness of numbers, CLOCK
42. COPYHAND	Presence of the two hands, CLOCK
43. COPYTIME	Presence of the two hands, set to ten after eleven, CLOCK
44. COPYSCOR	Total score, CLOCK
45. LDELCUE	Use of cue (0/1), LM
46. LDELTOTAL	Total number of story units recalled, Partial Score of LM test
47. LIMMTOTAL	Total number of story units recalled, LM Immediate Recall
48–53. AVTOT1-6	Total of each trial 1, 2, 3, 4, 5, 6, AVLT
54–59. AVERR1-6	Total intrusions of each trial 1, 2, 3, 4, 5, 6, AVLT
60. AVTOTB	Interference, AVLT
61. AVERRB	Total intrusions of List B, AVLT
62. AVDEL30MIN	30 minute delay, AVLT
63. AVDELERR1	Total intrusions, AVLT
64. AVDELTOT	Recognition, AVLT
65. AVDELERR2	Recognition errors, AVLT
66. DSPANFOR	Forward: Total Correct
67. DSPANFLTH	Forward: Length
68. DSPANBAC	Digit Span Backwards, Total Correct
69. DSPANBLTH	Backward: Length
70. DIGITSCOR	Digit Symbol
71. CATANIMSC	Category Fluency—Animals
72. CATANPERS	Category Fluency Animals—Perseverations
73. CATANINTR	Category Fluency (Animals)—Intrusions
74. CATVEGESC	Category Fluency Vegetables—Total Correct
75. CATVGPERS	Category Fluency (Vegetables) —Perseverations
76. CATVGINTR	Category Fluency (Vegetables)—Intrusions
77. TRAAERRCOM	Errors of commission, TMT
78. TRAAERROM	Errors of omission, TMT
79. TRAASCOR	Part A—time to complete, TMT
80. TRABERRCOM	Error of commission, TMT
81. TRABERROM	Error of omission, TMT
82. TRABSCOR	Part B—time to complete, TMT
83. BNTSPONT	Number of spontaneously given correct responses, Partial Score of BNT
84. BNTSTIM	Number of semantic cues given, Partial Score of BNT
85. BNTCSTIM	Number of correct responses following a semantic cue, Partial Score of BNT
86. BNTPHON	Number of phonemic cues given, Partial Score of BNT
87. BNTCPHON	Number of correct responses following a phonemic cue, Partial Score of BNT
88. BNTTOTAL	Total Number Correct (1 + 3)
89. ANARTERR	ANART Total Score (total number of errors)
90. Q1	Word Recall Task, ADAS-Cog
91. Q2	Following commands, ADAS-Cog
92. Q3	Constructional praxis, ADAS-Cog
93. Q4	Delayed Word Recall, ADAS-Cog
94. Q5	Naming objects and fingers, ADAS-Cog
95. Q6	Ideational practice, ADAS-Cog
96. Q7	Orientation, ADAS-Cog
97. Q8	Word recognition, ADAS-Cog
98. Q9	Remembering test instructions, ADAS-Cog
99. Q10	Comprehension of spoken and written language, ADAS-Cog
100. Q11	Word finding difficulty, ADAS-Cog
101. Q12	Language, ADAS-Cog
102. Q14	Number cancellation, ADAS-Cog
103. TOTAL11	Classic 70 points total, excluding Q4 and Q14, ADAS-Cog
104. TOTALMOD	85 points total, including Q4 and Q14, ADAS-Cog
105. GDSATIS	Are you basically satisfied with your life?, Partial Score of GDS
106. GDDROP	Have you dropped many of your activities and interests?, Partial Score of GDS
107. GDEMPTY	Do you feel that your life is empty?, Partial Score of GDS
108. GDBORED	Do you often get bored?, Partial Score of GDS
109. GDSPIRIT	Are you in good spirits most of the time?, Partial Score of GDS
110. GDAFRAID	Are you afraid that something bad is going to happen to you?, Partial Score of GDS
111. GDHAPPY	Do you feel happy most of the time?, Partial Score of GDS
112. GDHELP	Do you often feel helpless?, Partial Score of GDS
113. GDHOME	Do you prefer to stay at home, rather than going out and doing new things?, Partial Score of GDS
114. GDMEMORY	Do you feel you have more problems with memory than most?, Partial Score of GDS
115. GDALIVE	Do you think its wonderful to be alive now?, Partial Score of GDS
116. GDWORTH	Do you feel pretty worthless the way you are now?, Partial Score of GDS
117. GDENERGY	Do you feel full of energy?, Partial Score of GDS
118. GDHOPE	Do you feel that your situation is hopeless?, Partial Score of GDS
119. GDBETTER	Do you think that most people are better off than you are?, Partial Score of GDS
120. GDTOTAL	Total Score
121. FAQFINAN	Writing checks, paying bills, or balancing checkbook, Partial Score, FAQ
122. FAQFORM	Assembling tax records, business affairs, or other papers, Partial Score, FAQ
123. FAQSHOP	Shopping alone for clothes, household necessities, or groceries, Partial Score, FAQ
124. FAQGAME	Playing a game of skill such as bridge or chess, working on a hobby, Partial Score, FAQ
125. FAQBEVG	Heating water, making a cup of coffee, turning off the stove, Partial Score, FAQ
126. FAQMEAL	Preparing a balanced meal, Partial Score, FAQ
127. FAQEVENT	Keeping track of current events, Partial Score, FAQ
128. FAQTV	Paying attention to and understanding a TV program, book, or magazine, Partial Score, FAQ
129. FAQREM	Remembering appointments, family occasions, holidays, medications, Partial Score, FAQ
130. FAQTRAVL	Traveling out of the neighborhood, driving, or arranging to take public transportation, Partial Score, FAQ
131. FAQ total	Total Score, FAQ

**Table 2:** It shows the full list of neuropsychological features available from the ADNI database and those chosen by the neuropsychologists (in bold). The neuropsychologists adopted three criteria for selection: 1 = redundancy (or no high degree of independence, i.e., when the same cognitive process was assessed by different tests), 2 = overlap with CDR (which is used as gold standard for the classification), 3 = poor relevance to AD.

Status/domains/subdomains	Neuropsychological tests	Reason for exclusion
Global cognitive status/disease progression	**TOTALMOD (85 points total, including Q4 and Q14, ADAS)**	
	**TOTAL11 (Classic 70 points total, excluding Q4 and Q14, ADAS)**	
	**MMSCORE (total MMSE)**	
	ANARTERR, ANART Total Score (total number of errors)	3

Language	BNTSPONT (number of spontaneously given correct responses, Partial Score of BNT)	3
	BNTSTIM (number of semantic cues given, Partial Score of BNT)	3
	BNTCSTIM (number of correct responses following a semantic cue, Partial Score of BNT)	3
	BNTPHON (number of phonemic cues given, Partial Score of BNT)	3
	BNTCPHON (number of correct responses following a phonemic cue, Partial Score of BNT)	3
	**BNTTOTAL (Total Number Correct, BNT)**	
	CATANIMSC (Category Fluency—Animals, Total Correct)	1 (with CATVEGESC)
	CATANPERS (Category Fluency Animals—Perseverations)	1 (with CATVGPERS)
	CATANINTR (Category Fluency (Animals)—Intrusions)	1 (with CATANINTR)
	**CATVEGESC (Category Fluency Vegetables—Total Correct)**	
	**CATVGPERS (Category Fluency (Vegetables)—Perseverations)**	
	**CATANINTR (Category Fluency (Vegetables))**	
	**Q5 (naming objects and fingers, ADAS) **	
	**Q10 (comprehension of spoken and written language, ADAS)**	
	**Q11 (word finding difficulty)**	
	**Q12 (language, ADAS)**	
	MMWATCH	1 (with Q5)
	MMPENCIL	1 (with Q5)
	MMREPEAT	2
	**MMWRITE**	

Executive functioning	**TRABSCORE (Part B—time to complete, TMT)**	
	TRABERROROM (error of commission, TMT)	3
	TRABERROM (error of omission)	3

Memory and Learning	AVTOT3 (Total of each trial 1–3)	1 (with Q1)
	AVTOT4 (Total of each trial 1–4)	1 (with Q1)
	AVTOT5 (Total of each trial 1–5)	1 (with Q1)
	AVTOT6 (Total of each trial 1–6)	1 (with Q1)
	AVDELTOT (recognition, AVLT)	1 (with Q8)
	AVDEL30min (30-minute delay, AVLT)	1 (with Q4)
	**AVTOTB (interference)**	
	AVERR2 (total intrusions of trial 2)	1 (AVDELERR1–6)
	**AVDELERR1**–**6 (total intrusions, AVLT)**	
	MMSETrials	3
	MMBALLDL	1 (with Q4)
	MMFLAGDL	1 (with Q4)
	MMTREEDL	1 (with Q4)
	MMSEBALL	1 (with Q1)
	MMSEFLAG	1 (with Q1)
	MMSETREE	1 (with Q1)
	**LDELTOTAL (total number of story units recalled, Partial Score of LM test) **	
	**LIMMTOTAL (total number of story units recalled, LM Immediate Recall)**	
	**LDELCUE**	
	**Q1 (Word Recall Task, ADAS) **	
	**Q4 (Delayed Word Recall, ADAS) **	
	**Q8 (Word Recognition, ADAS) **	
	**Q9 (Remembering Test Instructions, ADAS)**	

Perceptual–motor coordination	**Q6 Ideational Praxis, ADAS** **Q3 Constructional Praxis, ADAS-Cog**	

Complex Attention	**TRAASCORE (Part A—time to complete, TMT)**	
	TRAAERRCOM, errors of commission, TMT	3
	TRAAERROM, errors of omission, TMT	3
	**DIGITSCORE (Digit Symbol)**	1 (with TRASCOR)
	**Q14 (Number Cancellation, ADAS)**	

Working memory	MMD	3
	MML	3
	MMR	3
	MMO	3
	MMW	3
	**DSPANFOR**	
	DSPANFLTH	3
	**DSPANBAC**	
	DSPANBLTH	3

Visuoconstructional reasoning	**CLOCKSCORE (Total Score, CLOCK)**	
	CLOCKSYM (symmetry of number placement, Partial Score of CLOCK)	3
	CLOCKNUM (correctness of numbers, Partial Score of CLOCK)	3
	CLOCKHAND (presence of the two hands, Partial Score of CLOCK)	3
	CLOCKTIME (presence of the two hands, set to ten after eleven, Partial Score of CLOCK)	3
	COPYSCORE (Total Score, CLOCK)	1 (with CLOCKSCOR)
	COPYNUM (correctness of numbers, Partial Score of CLOCK)	1 (with CLOCKNUM)
	COPYCIRC (approximately circular face, CLOCK)	1 (with CLOCKCIRC)
	COPYSYM (symmetry of number placement, Partial Score of CLOCK)	1 (with CLOCKSYM)
	COPYHAND (presence of the two hands, Partial Score of CLOCK)	1 (with CLOCKHAND)

Awarness (S/T Orientation)	MMDAY (what day of the week is it today? MMSE)	2
	MMDATE (what is today's date?, MMSE)	2
	MMYEAR (what is the year?, MMSE)	2
	MONTH (what is month are we in, MMSE)	2
	MMSEASON (what is season are we in?, MMSE)	3
	MMHOSPIT (what is the name of this hospital (clinic, place) MMSE)	2
	MMFLOR (what floor are we on?, MMSE)	3
	MMCITY (what town or city are we in?, MMSE)	2
	MMAREA (what county (district, borough, area) are we in?, MMSE)	2
	MMSTATE (what state are we in?, MMSE)	2
	Q7 (Orientation, ADAS)	2

Functional abilities	**FAQTOTAL (Total, FAQ)**	
	FAQFORM (assembling tax records, business affairs, or other papers, Partial score of FAQ)	2
	FAQBEVG (heating water, making a cup of coffee, turning off the stove. Partial Score, FAQ)	2
	FAQGAME (playing a game of skill such as bridge or chess, working on a hobby. Partial Score, FAQ)	2
	FAQFINAN (writing checks, paying bills, or balancing checkbook. Partial Score, FAQ)	2
	FAQMEAL (preparing a balanced meal, Partial score of FAQ)	3
	FAQTV (paying attention to and understanding a TV program, book, or magazine, Partial score of FAQ)	3
	FAQREM (remembering appointments, family occasions, holidays, medications, Partial score of FAQ)	2
	FAQSHOP (shopping alone for clothes, household necessities, or groceries, Partial Score of FAQ)	2
	FAQTRAVL (traveling out of the neighborhood, driving, or arranging to take public transportation, Partial score of FAQ)	2
	FAQEVENT (keeping track of current events, Partial Score of FAQ)	2

Depression	**GDTOTAL (Total score, GDS)**	
	GDHOPE (do you feel that your situation is hopeless?, Partial Score of GDS)	3
	GDDROP (have you dropped many of your activities and interests?, Partial Score of GDS)	3
	GDALIVE (do you think it is wonderful to be alive now?, Partial Score of GDS)	3
	GDHAPPY (do you feel happy most of the time?, Partial Score of GDS)	3
	GDWORTH (do you feel pretty worthless the way you are now?, Partial Score of GDS)	3
	GDENERGY (do you feel full of energy?, Partial Score of GDS)	3
	GDBETTER (do you think that most people are better off than you are?, Partial Score of GDS)	3
	GDSATIS (are you basically satisfied with your life?, Partial Score of GDS)	3
	GDEMPTY (is life empty?, Partial Score of GDS)	3
	GDBORED (do you often get bored?, Partial Score of GDS)	3
	GDSPIRIT (are you in good spirits most of the time?, Partial Score of GDS)	3
	GDAFRAID (are you afraid that something bad is going to happen to you?, Partial Score of GDS)	3
	GDHELP (do you often feel helpless?, Partial Score of GDS)	3
	GDHOME (do you prefer to stay at home, rather than going out and doing new things?, Partial Score of GDS)	3
	GDMEMORY (do you feel you have more problems with memory than most?, Partial Score of GDS)	3

**Table 3:** Performance of ML (accuracy, sensitivity, specificity, GM, and Dominance) in the classification of CDR = 1 versus CDR = 0 using linear, quadratic, Gaussian RBF, and Multilayer Perceptron kernels. Results were obtained using the computation-based feature reduction.

Kernel	Accuracy [mean ± std]^*∗*^	Sensitivity [mean ± std]^*∗*^	Specificity [mean ± std]^*∗*^	Geometric Mean [mean ± std]^*∗*^	Dominance [mean ± std]^*∗*^
Linear	0.91 ± 0.07	0.89 ± 0.15	0.92 ± 0.08	0.90 ± 0.09	−0.03 ± 0.17
Quadratic	0.91 ± 0.07	0.87 ± 0.16	0.92 ± 0.08	0.89 ± 0.10	−0.05 ± 0.18
Gaussian RBF	0.92 ± 0.07	0.90 ± 0.14	0.93 ± 0.08	0.91 ± 0.09	−0.03 ± 0.16
Multilayer Perceptron	0.91 ± 0.07	0.87 ± 0.16	0.93 ± 0.08	0.90 ± 0.10	−0.06 ± 0.18

^*∗*^Averaged across 10 rounds of the nested CV and across 100 iterations.

**Table 4:** Performance of ML (accuracy, sensitivity, specificity, GM, and Dominance) in the classification of CDR = 0.5 versus CDR = 0 using linear, quadratic, Gaussian RBF, and Multilayer Perceptron kernels. Results were obtained using the computation-based feature reduction.

Kernel	Accuracy [mean ± std]^*∗*^	Sensitivity [mean ± std]^*∗*^	Specificity [mean ± std]^*∗*^	Geometric Mean [mean ± std]^*∗*^	Dominance [mean ± std]^*∗*^
Linear	0.86 ± 0.07	0.85 ± 0.10	0.87 ± 0.10	0.86 ± 0.07	−0.01 ± 0.15
Quadratic	0.86 ± 0.07	0.85 ± 0.11	0.88 ± 0.09	0.86 ± 0.07	−0.03 ± 0.15
Gaussian RBF	0.86 ± 0.07	0.85 ± 0.10	0.87 ± 0.10	0.86 ± 0.07	−0.02 ± 0.15
Multilayer Perceptron	0.85 ± 0.07	0.83 ± 0.12	0.87 ± 0.10	0.85 ± 0.07	−0.04 ± 0.16

^*∗*^Averaged across 10 rounds of the nested CV and across 100 iterations.

**Table 5:** Performance of ML (accuracy, sensitivity, specificity, GM, and Dominance) in the classification of CDR = 1 versus CDR = 0.5 using linear, quadratic, Gaussian RBF, and Multilayer Perceptron kernels. Results were obtained using the computation-based feature reduction.

Kernel	Accuracy [mean ± std]^*∗*^	Sensitivity [mean ± std]^*∗*^	Specificity [mean ± std]^*∗*^	Geometric Mean [mean ± std]^*∗*^	Dominance [mean ± std]^*∗*^
Linear	0.65 ± 0.12	0.59 ± 0.22	0.67 ± 0.16	0.60 ± 0.16	−0.09 ± 0.30
Quadratic	0.65 ± 0.12	0.59 ± 0.23	0.67 ± 0.16	0.60 ± 0.16	−0.07 ± 0.30
Gaussian RBF	0.64 ± 0.12	0.61 ± 0.23	0.65 ± 0.16	0.60 ± 0.16	−0.05 ± 0.31
Multilayer Perceptron	0.63 ± 0.12	0.62 ± 0.24	0.63 ± 0.17	0.60 ± 0.15	0 ± 0.33

^*∗*^Averaged across 10 rounds of the nested CV and across 100 iterations.

**Table 6:** Performance of ML (accuracy) in the inner and outer loops for each of the 10 rounds (averaged across 100 iterations). Results are reported for CDR = 1 versus CDR = 0, CDR = 0.5 versus CDR = 0, and CDR = 1 versus CDR = 0.5 using a linear kernel and the computation-based feature reduction.

Round	CDR = 1 versus CDR = 0		CDR = 0.5 versus CDR = 0		CDR = 1 versus CDR = 0.5	
	Inner loop accuracy ^*∗*^	Outer loop accuracy ^*∗*^	Inner loop accuracy ^*∗*^	Outer loop accuracy ^*∗*^	Inner loop accuracy ^*∗*^	Outer loop accuracy ^*∗*^
1	0.95 ± 0.02	0.91 ± 0.06	0.87 ± 0.03	0.87 ± 0.07	0.75 ± 0.03	0.64 ± 0.14
2	0.95 ± 0.02	0.90 ± 0.07	0.87 ± 0.03	0.85 ± 0.07	0.75 ± 0.03	0.65 ± 0.12
3	0.95 ± 0.02	0.91 ± 0.07	0.87 ± 0.02	0.85 ± 0.07	0.75 ± 0.03	0.65 ± 0.12
4	0.95 ± 0.02	0.92 ± 0.07	0.87 ± 0.03	0.87 ± 0.07	0.75 ± 0.03	0.65 ± 0.10
5	0.95 ± 0.02	0.92 ± 0.06	0.87 ± 0.03	0.85 ± 0.07	0.76 ± 0.04	0.62 ± 0.13
6	0.95 ± 0.02	0.91 ± 0.07	0.87 ± 0.03	0.87 ± 0.06	0.75 ± 0.03	0.66 ± 0.13
7	0.95 ± 0.02	0.91 ± 0.07	0.87 ± 0.02	0.86 ± 0.06	0.75 ± 0.03	0.66 ± 0.10
8	0.95 ± 0.02	0.91 ± 0.07	0.86 ± 0.03	0.86 ± 0.06	0.75 ± 0.03	0.64 ± 0.11
9	0.95 ± 0.03	0.90 ± 0.08	0.87 ± 0.03	0.85 ± 0.07	0.75 ± 0.03	0.65 ± 0.11
10	0.95 ± 0.03	0.92 ± 0.07	0.87 ± 0.03	0.86 ± 0.08	0.76 ± 0.03	0.64 ± 0.12

Total mean^*∗∗*^	0.95 ± 0.01	0.91 ± 0.07	0.87 ± 0.01	0.86 ± 0.07	0.75 ± 0.01	0.65 ± 0.12

^*∗*^mean ± std averaged across 100 iterations; ^*∗∗*^mean ± std averaged across 100 iterations and 10 rounds.

**Table 7:** Performance of ML (accuracy, sensitivity, specificity, GM, and Dominance) in the classification of CDR = 1 versus CDR = 0.5, CDR = 0.5 versus CDR = 0 and CDR = 1 versus CDR = 0.5. Results were obtained using the feature reduction guided by the neuropsychologists.

Level of impairment	Accuracy [mean ± std]^*∗*^	Sensitivity [mean ± std]^*∗*^	Specificity [mean ± std]^*∗*^	Geometric Mean [mean ± std]^*∗*^	Dominance [mean ± std]^*∗*^
CDR = 1 vs CDR = 0	0.96 ± 0.04	0.95 ± 0.10	0.97 ± 0.05	0.96 ± 0.06	−0.03 ± 0.11
CDR = 0.5 vs CDR = 0	0.86 ± 0.07	0.84 ± 0.10	0.89 ± 0.09	0.86 ± 0.07	−0.05 ± 0.13
CDR = 1 vs CDR = 0.5	0.69 ± 0.10	0.67 ± 0.21	0.70 ± 0.13	0.67 ± 0.13	−0.03 ± 0.26

^*∗*^Across 10 rounds of the nested CV and across 100 iterations.

**Table 8:** Performance of ML (accuracy) in the inner and outer loops for each of the 10 rounds (averaged across 100 iterations). Results are reported for CDR = 1 versus CDR = 0, CDR = 0.5 versus CDR = 0, and CDR = 1 versus CDR = 0.5 using a linear kernel and the feature reduction guided by the neuropsychologists.

Round	CDR = 1 versus CDR = 0		CDR = 0.5 versus CDR = 0		CDR = 1 versus CDR = 0.5	
	Inner loop accuracy ^*∗*^	Outer loop accuracy ^*∗*^	Inner loop accuracy ^*∗*^	Outer loop accuracy ^*∗*^	Inner loop accuracy ^*∗*^	Outer loop accuracy ^*∗*^
1	1 ± 0.01	0.96 ± 0.05	0.91 ± 0.02	0.87 ± 0.07	0.81 ± 0.03	0.70 ± 0.11
2	1 ± 0.01	0.96 ± 0.05	0.91 ± 0.02	0.85 ± 0.06	0.81 ± 0.03	0.70 ± 0.10
3	1 ± 0.01	0.96 ± 0.05	0.91 ± 0.02	0.86 ± 0.06	0.81 ± 0.03	0.69 ± 0.10
4	0.99 ± 0.01	0.97 ± 0.04	0.91 ± 0.02	0.85 ± 0.07	0.81 ± 0.03	0.69 ± 0.09
5	0.99 ± 0.01	0.97 ± 0.04	0.91 ± 0.02	0.85 ± 0.06	0.81 ± 0.04	0.69 ± 0.10
6	0.99 ± 0.01	0.96 ± 0.05	0.91 ± 0.02	0.86 ± 0.06	0.81 ± 0.03	0.70 ± 0.11
7	0.99 ± 0.01	0.97 ± 0.04	0.91 ± 0.02	0.86 ± 0.07	0.80 ± 0.04	0.70 ± 0.11
8	0.99 ± 0.01	0.96 ± 0.04	0.91 ± 0.02	0.87 ± 0.05	0.81 ± 0.04	0.69 ± 0.10
9	0.99 ± 0.01	0.97 ± 0.04	0.91 ± 0.02	0.86 ± 0.07	0.80 ± 0.04	0.71 ± 0.10
10	0.99 ± 0.01	0.97 ± 0.05	0.91 ± 0.02	0.86 ± 0.07	0.81 ± 0.04	0.67 ± 0.11

Total mean^*∗∗*^	0.99 ± 0.01	0.96 ± 0.04	0.91 ± 0.00	0.86 ± 0.07	0.81 ± 0.01	0.69 ± 0.10

^*∗*^mean ± std averaged across 100 iterations; ^*∗∗*^mean ± std averaged across 100 iterations and 10 rounds.

**Table 9:** Top 10 features most frequently found as best predictors across all 10 rounds and all 100 iterations using the FDR feature reduction.

Level of impairment	Features	Frequency^*∗*^
CDR = 1 versus CDR = 0	(1) LDELTOTAL (LM)	71%
	(2) TOTALMOD (ADAS)	10%
	(3) LIMMTOTAL (LM)	4%
	(4) FAQTOTAL (FAQ)	4%
	(5) Q4 (ADAS)	4%
	(6) AVTOT5 (AVLT)	3%
	(7) AVTOT4 (AVLT)	1%
	(8) Q1 (ADAS)	0.8%
	(9) AVDEL30MIN (AVLT)	0.6%
	(10) TOTAL11 (ADAS)	0.5%

CDR = 0.5 versus CDR = 0	(1) LDELTOTAL (LM)	91%
	(2) Q4 (ADAS-Cog)	22%
	(3) LIMMTOTAL (LM)	15%
	(4) TOTALMOD (ADAS-Cog)	12%
	(5) GDHOPE (GDS)	6%
	(6) MMD (MMSE)	2%
	(7) MMSCORE (MMSE)	0.3%
	(8) AVTOT4 (AVLT)	0.1%
	(9) CATVEGESC (Semantic Fluency Test)	0.1%
	(10) TOTAL11 (ADAS)	0.1%

CDR = 1 versus CDR = 0.5	(1) FAQTOTAL (FAQ)	31%
	(2) TOTALMOD (ADAS-Cog)	22%
	(3) AVTOT5 (AVLT)	10%
	(4) FAQFORM (FAQ) (5) Q1 (ADAS-Cog)	6% 6%
	(6) FAQREM (FAQ)	6%
	(7) TOTAL11 (ADAS)	5%
	(8) CLOCKSCOR (CLOCK Test)	4%
	(9) CATVEGESC (Semantic Fluency Test)	4%
	(10) Q8 (ADAS)	4%

^*∗*^Across 10 rounds of the nested CV and across 100 iterations.

**Table 10:** Top 10 features most frequently found as best predictors across all 10 rounds and all 100 iterations using the features chosen by the neuropsychologists.

Level of impairment	Features	Frequency^*∗*^
CDR = 1 versus CDR = 0	(1) LDELTOTAL (Logical Memory Test)	80%
	(2) TOTALMOD (ADAS)	50%
	(3) FAQ total (FAQ)	29%
	(4) TOTAL11 (ADAS)	18%
	(5) CATVEGESC (Semantic Fluency Test)	13%
	(6) Q4 (ADAS)	13%
	(7) LIMMTOTAL (Logical Memory)	9%
	(8) Q8 (ADAS)	5%
	(9) MMSCORE (MMSE)	5%
	(10) Q1 (ADAS)	3%

CDR = 0.5 versus CDR = 0	(1) FAQ total (FAQ)	81%
	(2) LDELTOTAL (Logical Memory Test)	77%
	(3) Q4 (ADAS)	44%
	(4) TOTALMOD (ADAS)	39%
	(5) CATVEGESC (Semantic Fluency Test)	36%
	(6) LIMMTOTAL (Logical Memory)	30%
	(7) MMSCORE (MMSE)	30%
	(8) Q8 (ADAS)	23%
	(9) TOTAL11 (ADAS)	19%
	(10) Q1 (ADAS)	19%

CDR = 1 versus CDR = 0.5	(1) FAQ total (FAQ)	82%
	(2) CLOCKSCOR (CLOCK Test)	36%
	(3) Q8 (ADAS)	35%
	(4) LDELCUE (Logical Memory Test)	33%
	(5) TOTAL11 (ADAS)	30%
	(6) Q4 (ADAS)	29%
	(7) TOTALMOD (ADAS)	22%
	(8) LDELTOTAL (Logical Memory Test)	20%
	(9) CATVEGESC (Semantic Fluency Test)	19%
	(10) Q1 (ADAS)	18%

^*∗*^Across 10 rounds of the nested CV and across 100 iterations.
